# Development of Magnetic Porous Polymer Composite for Magnetic Solid Phase Extraction of Three Fluoroquinolones in Milk

**DOI:** 10.3390/foods13162511

**Published:** 2024-08-12

**Authors:** Zhendong Yu, Tiantian Xu, Shumin Lin, Shuxuan Liang

**Affiliations:** 1 Key Laboratory of Analytical Science and Technology of Hebei Province, College of Chemistry and Materials Science, Hebei University, Baoding 071002, China; zhendong1205@126.com (Z.Y.); xtiantian202202@163.com (T.X.); lsmnmg@126.com (S.L.); 2 College of Public Health, Hebei University, Baoding 071002, China

**Keywords:** magnetic solid phase extraction, metal organic framework, ProElut polystyrene polymers, milk, fluoroquinolones

## Abstract

In this study, a magnetic porous polymer composite with both hydrophilic and hydrophobic groups was synthesized for magnetic solid phase extraction (MSPE) of milk substrates. Optimization was conducted on various parameters, including adsorption dose, solution pH, adsorption time, and some elution conditions. Coupled with a high-performance liquid chromatography fluorescence detector, a novel MSPE method for determination of norfloxacin (NFX), ciprofloxacin (CIP), and enrofloxacin (ENR) in milk was developed based on magnetic metal organic framework polystyrene polymer (Fe_3_O_4_@MOF@PLS) as adsorbent. The Fe_3_O_4_@MOF@PLS exhibited significantly improved adsorption performance compared to MOF and PLS. Under optimized experimental conditions, the method exhibited good linearity for the three fluoroquinolones (FQs) in the range of 0.5–1000 μg/kg, with limit of detections (LODs) ranging from 0.21 to 1.33 μg/kg, and limit of quantitations (LOQs) from 0.71 to 4.42 μg/kg. The relative standard deviation (RSD) for the three FQs were 3.4–8.8%. The recoveries of three FQs in milk samples ranged from 84.2% to 106.2%. This method was successfully applied to the detection of three FQs in 20 types of milk, demonstrating its simplicity, speed, and effectiveness in analyte enrichment and separation. The method presented advantages in adsorbent dosage, adsorption time, LODs, and LOQs, making it valuable for the analysis and detection of FQs in milk.

## 1. Introduction

In the past hundred years, antibiotics have played a crucial role in preventing and treating bacterial infections in both human and veterinary medicine, as well as in the advancement of animal husbandry and aquaculture [1,2,3]. Fluoroquinolones (FQs) are a significant class of antibiotics, known for their broad-spectrum antibacterial properties, acting by inhibiting bacterial DNA gyrase and interfering with DNA replication to achieve a bactericidal effect [4,5]. However, the widespread use of FQs, has resulted in the presence of excessive antibiotic residues in surface water, soil, and various animal products, such as fish, shrimp, and dairy items, posing potential public health risks even at low concentrations [6,7,8]. To safeguard consumer safety, some countries and organizations have established regulatory limits on FQs in food [9,10]. For instance, China, the European Union, and Japan have set maximum residue limits (MRLs) for enrofloxacin (ENR) in milk at 100, 100, and 50 μg/kg, respectively [11], with ciprofloxacin (CIP) having a MRL of 100 μg/kg [12,13]. Long-term consumption of animal-derived products containing excessive veterinary drug residues can lead to drug resistance and various toxic side effects, such as liver injury, thrombus, and damage to the central nervous system [10,14]. Therefore, it is necessary to develop a simple, rapid, and selective method to monitor the residue level of FQs.

The commonly used detection methods of FQs are capillary electrophoresis [15], sensor method [16], high-performance liquid chromatography (HPLC) [17], high-performance liquid chromatography–tandem mass spectrometry [18], and so on. HPLC is the most commonly used method for the detection of antibiotic residues due to its excellent separation and detection capabilities, simplicity, rapidity, low limit of detection (LOD), and good reproducibility [19]. In HPLC, different polarity values of the solvents are utilized to separate and detect components using a high-pressure pump, with analysis and detection based on suitable detectors and different retention times [20]. Commonly used detectors include the fluorescence detector (FLD), ultraviolet detector, and diode array detector, with FLD offering high sensitivity and the lowest detection concentration [21]. Given the complexity of food sample matrices and the low concentrations of FQs in food, there is an increasing need to develop highly sensitive enrichment and separation methods for sample pretreatment to accurately and rapidly detect trace antibiotics in food.

At present, the commonly used pretreatment methods in drug analysis are liquid–liquid extraction [22], solid-phase extraction (SPE) [23], magnetic solid-phase extraction (MSPE) [24,25,26], and solid-phase microextraction [27]. Traditional SPE columns face limitations due to the diffusion rate of the material, resulting in long equilibrium times. In contrast, MSPE utilizes micron or nanometer particles that disperse quickly throughout the sample system, enhancing contact with the target and accelerating the adsorption process and efficiency. This allows for savings in the amount of adsorbent used. Additionally, magnetic particles in MSPE can be easily separated and collected from the system under the influence of an external magnetic field, eliminating the need for a complex filtration or centrifugation process and reducing adsorbent loss [28]. Hong et al. established ionic liquid-MSPE technology for HPLC extraction of FQs in honey and milk, showing good linearity, low limit of quantification (LOQ), and desirable recovery of FQs [29]. Notably, the magnetic adsorbent plays a crucial role in MSPE technology, highlighting the importance of developing new adsorbents and establishing simpler, faster, and more cost-effective sample pretreatment and analysis methods.

A metal organic framework (MOF) is a porous coordination crystal structure, in which a metal cluster is connected with organic ligands through coordination bonds. Different pore sizes are designed according to the types of molecules to improve selectivity and reusability [30,31]. With their large specific surface area, adjustable pore size, good stability, and controllable chemical properties, MOFs hold significant promise in separation science [32,33]. MOFs are a kind of scaffold material suitable for magnetic nanoparticles, and have been widely used as efficient and multi-functional MSPE adsorbents [34]. MIL series materials are representative materials of interest in the field of MOFs. MIL-101 is widely used in the study of adsorption and removal of antibiotics because of its ultra-high specific surface area, good heat resistance, and chemical properties. For example, Lian et al. synthesized Fe_3_O_4_@Cys@MIL-125-NH_2_ and established a MSPE-UHPLC method for the detection of five FQs in rivers [35]. Guo et al. introduced a stable mesoporous MOF, MIL-101 (Cr)-SO_3_H, functionalized with polar-SO_3_H groups to efficiently adsorb and remove FQs from water, demonstrating high adsorption capacity and excellent reusability [36]. These studies underscore the potential of MIL series materials in the adsorption and sample pretreatment of FQs.

**Scheme 1:**
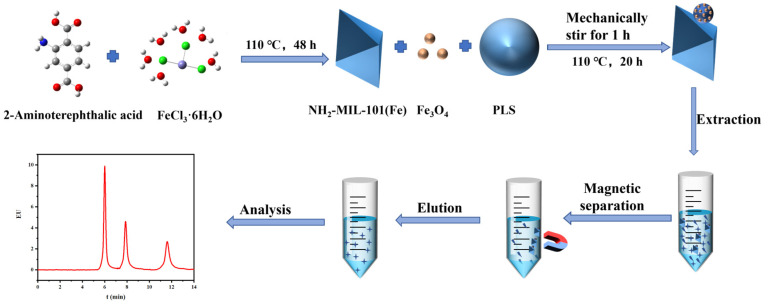
Synthesis of Fe_3_O_4_@MOF@PLS and its application as MSPE adsorbent for determination of three kinds of FQs by HPLC.

ProElut polystyrene polymers (PLS) are spherical particles of polystyrene and divinylbenzene copolymers containing hydrophilic groups, which have a good retention rate for polar and non-polar compounds. In this study, magnetic composite material of PLS and NH_2_-MIL-101(Fe) (Fe_3_O_4_@MOF@PLS) was successfully synthesized for the first time by leveraging the advantageous adsorption capacity and easy functionalization of MOF materials, combined with the versatility and stability of PLS. Then, using this material as an MSPE adsorbent, a new method for simultaneous determination of NFX, CIP, and ENR in milk samples by Fe_3_O_4_@MOF@PLS-MSPE-HPLC-FLD was established by optimizing a series of conditions. Scheme 1 shows the preparation of Fe_3_O_4_@MOF@PLS and the MSPE process for FQ detection. Through the comparative experiments and a series of basic characterization of the adsorption material, it is proved that the material has good practical application ability. The method has been successfully applied to the determination of three FQs in milk samples. 

## 2. Materials and Methods

### 2.1. Chemical Reagents and Materials

Ferric chloride hexahydrate (FeCl_3_·6H_2_O), sodium citrate (C_6_H_5_Na_3_O_7_·2H_2_O), ammonium acetate (CH_3_COONH_4_), acetic acid (HAc), sodium hydroxide (NaOH), ammonia (NH_3_·H_2_O), N,N-dimethylacetamide (DMF), 2-aminoterephthalic acid, methanol, absolute ethanol, ethylene glycol, acetonitrile (HPLC-grade), norfloxacin (NFX, ≥98%), CIP (≥98%), and ENR (≥98%) were procurement from Shanghai Aladdin Chemical Co., Ltd. (Shanghai, China). All chemicals were of analytical grade unless otherwise specified and all aqueous solutions were prepared with ultrapure water. PLS (DiKMA, Shanghai, China, ≥99%) had a particle size of 50 μm and the full aperture of 8 nm. Ultrapure water (18.25 mΩ) was prepared by a molecular purification system (Chongqing, China) and used throughout the study.

### 2.2. Instrumental Analysis

The instrumental analysis was carried out using a HPLC system (Waters Corporation, Milford, CT, USA), which was equipped with two delivery pumps (model 1525 binary HPLC pump) and a fluorescence detector (model 2475 detector). A baseline C18 column (5 μm, 4.6 mm i.d. × 25 cm long, Waters Corporation, Milford, USA) was kept at 30 °C for separation of analytes. The optimized mobile phase was composed of acetonitrile and solution containing 0.2% acetic acid (*v*/*v* = 8/2) and the flow rate was maintained at 1.0 mL/min. Sample injection volume was 10 μL. The detection wavelength was set to 450 nm.

### 2.3. Preparation of the MSPE Material

#### 2.3.1. Preparation of Fe_3_O_4_

Fe_3_O_4_ nanospheres were synthesized using a solvothermal reaction. Typically, 1.35 g FeCl_3_·6H_2_O, 0.4 g of C_6_H_5_Na_3_O_7_·2H_2_O and 3.85 g of CH_3_COONH_4_ were mixed in a 100 mL conical flask, 70 mL of ethylene glycol was added to it, and the solution was dispersed evenly by ultrasound for 5 min. The mixed reactants were electromagnetically stirred at 50 °C for 1 h and shifted to a Teflon autoclave with a volume of 100 mL. The autoclave was heated to 200 °C and kept at this temperature for 16 h. The product was washed with alcohol three times and dried in a 60 °C vacuum drying oven for 12 h for further use. 

#### 2.3.2. Preparation of MOF

Firstly, 3.12 g FeCl_3_·6H_2_O and 35 mL DMF were mixed in a 100 mL conical flask. Then, 1.04 g 2-aminoterephthalic acid and 35 mL DMF were mixed into another 100 mL conical flask. The above two systems were mixed and sonicated for 5 min, and then transferred to a 100 mL polytetrafluoroethylene high-pressure reactor. The mixture was heated at 110 °C and lasted for 48 h. The resulting product was washed 4 times with DMF and absolute ethanol, respectively. Finally, MOF was obtained by drying at 60 °C in an oven for 12 h.

#### 2.3.3. Preparation of Fe_3_O_4_@MOF@PLS

Firstly, 20 mg of PLS, 35 mg of MOF, and 40 mg of Fe_3_O_4_ in a ratio of 1:1.75:2 were mixed into a 100 mL conical flask; then, 40 mL anhydrous ethanol was added and mechanically stirred at room temperature for 1 h. Then, the mixture was transferred to a 100 mL polytetrafluoroethylene high-pressure reactor and reacted at 110 °C for 20 h. Whereafter the product was washed 4 times with absolute ethanol. Finally, it was dried at 60 °C for 8 h to obtain the final product.

The morphology, structure, and property of Fe_3_O_4_@MOF@PLS were characterized by Fourier transform infrared spectroscopy (FT-IR) (Nicolet, Madison, WI, USA), scanning electron microscope (SEM) (Hitachi, Tokyo, Japan), X-ray diffractometer (XRD) (Bruker, Karlsruhe, Selb, Germany), and thermogravimetric analyzer with differential scanning calorimeter (TGA-DSC) (Netzsch, Selb, Germany).

### 2.4. Sample Collection and Pretreatment

A total of 20 milk samples were purchased from a supermarket in the locality in Baoding (Hebei Province, China). The milk samples were made up of five different batches of four brands. Initially, it was essential to conduct a pre-treatment step involving the precipitation of protein from the samples. Proteins significantly interfere with the adsorption experiments. Firstly, the complex matrix can compromise detection accuracy and lead to contamination of the instruments, thereby affecting the detection limit. Additionally, the viscosity of the protein can influence the mass transfer of antibiotics in solution, which, in turn, impacts both the extraction and adsorption efficiency. Consequently, the protein precipitation process was carried out through the following steps. Firstly, 2 mL homogeneous sample and 10 mL acetonitrile were put into the cover centrifugal pipe. Then, the centrifugal tube was stirred vigorously on a vortex mixer for 1 min and centrifuged through centrifuge at 6000 rpm for 5 min. Subsequently, the milk residue was treated with 5 mL of acetonitrile and 2.5 mL of PBS with a pH of 3. The supernatant was combined on a rotating evaporator at 50 °C and concentrated until nearly dry. Finally, 2 mL 0.2% acetic acid aqueous solution was added for resolution for the next step.

### 2.5. Procedure of MSPE

Firstly, 5 mg Fe_3_O_4_@MOF@PLS was added to the prepared solution, and the pH of the solution was adjusted to 7.0 with KH_2_PO_4_-K_2_HPO_4_ buffer solution. The sample was adsorbed by shaking at 30 °C for 20 min. Then, a magnet was used as an external magnetic field, Fe_3_O_4_@MOF@PLS was adsorbed at the bottom of the centrifuge tube, the upper liquid was discarded, and 5 mL 0.1% ammonia acetonitrile solution as eluent was added to the centrifuge tube. The target substance was eluted by ultrasound for 5 min, then an external magnet was used to adsorb the material at the bottom of the centrifuge tube, retain the upper liquid, and blow nitrogen to near dryness under mild nitrogen flow. Finally, it was dissolved in 2 mL aqueous solution containing 0.2% acetic acid and passed through 0.22 μm organic filter membrane for the next HPLC analysis.

### 2.6. Adsorption Experiment

The adsorption isotherm experiment was carried out using the following steps. Firstly, 5 mg of Fe_3_O_4_@MOF@PLS composite was added into 50 mL standard solution with varying concentrations (10–50 mg/L). The solutions were successively shaken at 150 rpm/min at 30, 40, and 50 °C for 7 h until the condition of adsorption equilibrium was met. The supernatants were detected using a UV–visible spectrophotometer (UV–vis) after the sorbents had been separated. The adsorption capacity was calculated according to Equation (1).

(1) $$q_{e}=\frac{\left(C_{0}-C_{e}\right)\times V}{m}$$

where q_e_ (mg/g) is the adsorption capacity of the adsorbent, C_0_ (mg/L) is the initial concentration of FQs, C_e_ (mg/L) is the final concentration of FQs, V (mL) is the solution volume, and m (mg) is the weight of the adsorbent.

The selectivity of synthesized Fe_3_O_4_@MOF@PLS was confirmed by conducting the adsorption experiment using different antibiotics, including tetracyclines, aminoglycosides, β-lactams, and FQs (dosage of Fe_3_O_4_@MOF@PLS and Fe_3_O_4_@PLS: 5 mg; concentration antibiotic solution: 20 mg/L; volume: 50 mL).

## 3. Results and Discussion

### 3.1. Adsorption Performance and Extraction Effect of Composite Adsorbent

#### 3.1.1. Adsorption Performance

The Freundlich and Langmuir isotherm models were employed to simulate the adsorption isotherms. The adsorption of Fe_3_O_4_@MOF@PLS demonstrates a closer alignment with the Langmuir model than with the Freundlich model, as evidenced by the fitting results presented in Figure S1 and Table S1. The maximum adsorption capacities for NFX, CIP, and ENR were measured at 105.0, 90.7, and 104.0 mg/g, respectively. Adsorption experiments conducted at varying oscillation temperatures indicate that increased temperature and concentration contribute positively to the enhancement of adsorption capacity (Figure S1). As illustrated in Figure S2, the synthesized Fe_3_O_4_@MOF@PLS exhibits poor adsorption capacity for tetracycline, which shares the same β-diketone structure, and also demonstrates low adsorption capacity for the other two antibiotics commonly utilized in livestock. This finding confirmed that the material synthesized in this study possesses good adsorption selectivity for NFX, CIP, and ENR, with minimal interference from other antibiotics presented in milk samples. Furthermore, it was observed that the composite materials exhibit a greater adsorption capacity compared to Fe_3_O_4_@PLS, thereby affirming their suitability for the detection of these three antibiotics.

#### 3.1.2. Determination of Adsorption Extraction Effect of Composite Adsorbent

**Figure 1:**
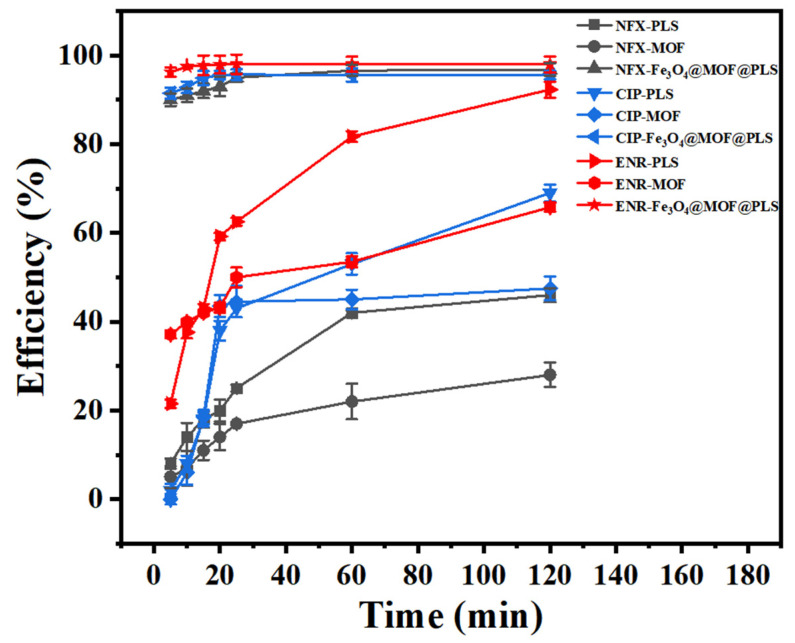
Comparison of adsorption effects of Fe_3_O_4_@MOF@PLS, MOF, and PLS on NFX, CIP and ENR of 50 μg/L.

In order to investigate the adsorption effect of the Fe_3_O_4_@MOF@PLS on NFX, CIP, and ENR, the adsorption capacities of three kinds of adsorbents were compared in this experiment. The results presented in Figure 1 indicate that the adsorption efficiency of Fe_3_O_4_@MOF@PLS for all three FQs exceed 90%, surpassing the performance of PLS and MOF. This enhanced efficiency may be attributed to the presence of both hydrophilic and hydrophobic groups in the material, which facilitates a more rapid and effective binding with the three FQs.

### 3.2. Characterization of Fe_3_O_4_@MOF@PLS

**Figure 2:**
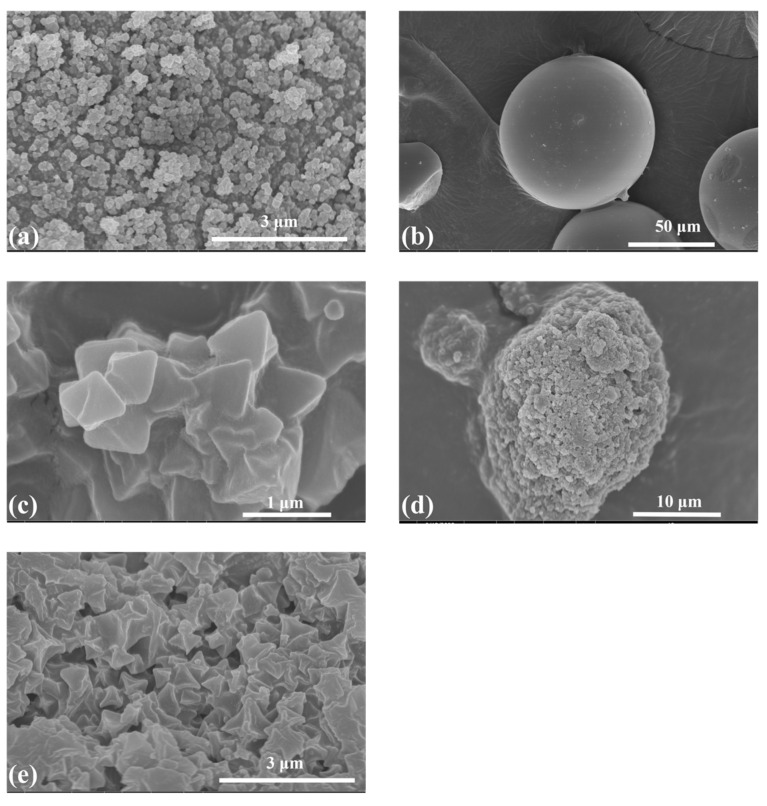
SEM images of: (**a**) Fe_3_O_4_, (**b**) PLS, (**c**) MOF, (**d**) Fe_3_O_4_@PLS, and (**e**) Fe_3_O_4_@MOF@PLS.

The morphologies of Fe_3_O_4_, PLS, MOF, and Fe_3_O_4_@MOF@PLS were studied by SEM. As shown in Figure 2a, it is evident that most ferric oxide nanoparticles exhibit a regular spherical structure and uniform particle size distribution. PLS is characterized as a polymerized microsphere with a smooth surface (Figure 2b). Upon the growth of MOF on the surface of ferric oxide nanoparticles, Fe_3_O_4_@MOF with an octahedral shape and good porosity is formed (Figure 2c). The SEM images in Figure 2d,e and the former study [37] reveal the composite surface, showing that the surface is composed of spherical PLS embedded with Fe_3_O_4_ and NH_2_-MIL-101 (Fe) with an octahedron shape.

**Figure 3:**
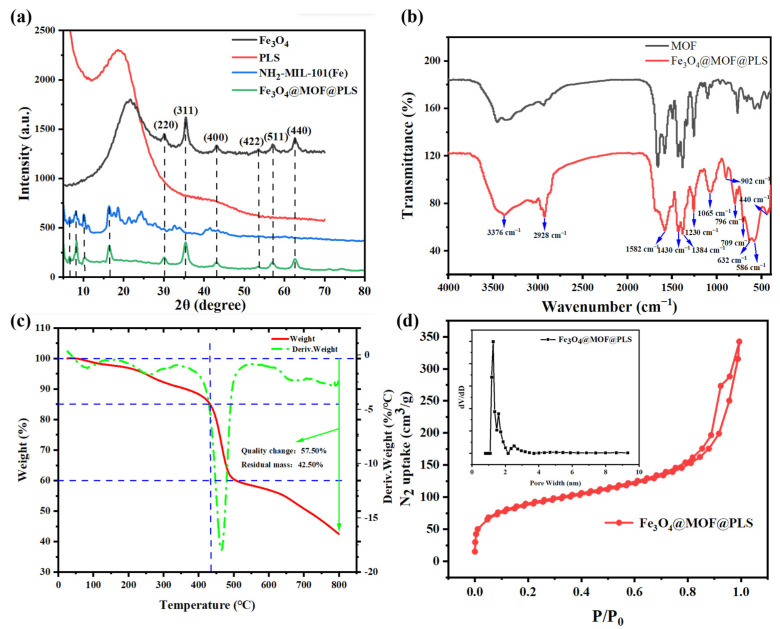
(**a**) X-ray diffraction patterns of Fe_3_O_4_, PLS, MOF, and Fe_3_O_4_@MOF@PLS; (**b**) infrared spectra of MOF and Fe_3_O_4_@MOF@PLS; (**c**) thermogravimetric map of Fe_3_O_4_@MOF@PLS; (**d**) N_2_ adsorption and desorption isotherms of Fe_3_O_4_@MOF@PLS.

To further demonstrate the successful synthesis of Fe_3_O_4_@MOF@PLS, XRD and FT-IR analyses were conducted to characterize the crystal structure and molecular structure. As shown in Figure 3a, PLS has no characteristic diffraction peak, indicating that it has an amorphous structure. However, Fe_3_O_4_@MOF@PLS exhibits prominent diffraction peaks at 5.11°, 8.21°, 10.18°, and 16.34°, corresponding to MOF. Additionally, diffraction peaks at 30.1°, 35.5°, 43.1°, 53.7°, 57.1°, and 62.5° match the crystal planes (220), (311), (400), (422), (511), and (440) of Fe_3_O_4_, confirming the presence of Fe_3_O_4_ and MOF in Fe_3_O_4_@MOF@PLS and validating the successful synthesis.

In Figure 3b, the broad band at approximately 3376 cm^−1^ is related to the stretching vibration of N-H in amino terylene acid, and the energy peaks at 709–901 cm^−1^ are also related to the out-of-plane C-H bending vibration of the H_2_BDC benzene ring. Two strong peaks at 1384 cm^−1^ and 1582 cm^−1^ are attributed to asymmetric and symmetric stretching vibrations of C-O in the carboxyl group, respectively, confirming the presence of dicarboxylic acid connectors in the material. An asymmetric stretching vibration of C-H in vinyl is found at 2928 cm^−1^ and the stretching vibration at 1429 cm^−1^ is due to the presence of C-N bonds in the pyrrolidone group. In addition, the apparent peaks at 440–632 cm^−1^ are the stretching vibration of Fe-O. The peak at 586 cm^−1^ is attributed to the characteristic peak of PLS (Figure S3a). These findings collectively support the successful synthesis of Fe_3_O_4_@MOF@PLS, as reported in references [38,39,40,41,42].

The pyrolytic characterization of Fe_3_O_4_@MOF@PLS was studied by TG-DSC. As shown in Figure 3c, the weight loss before 100 °C is due to the evaporation of water present in the composite material. Subsequently, in the temperature range of 100–450 °C, the loss of structural water and some weak groups causes a continuous decrease in weight. Up to 450 °C, only approximately 15% weight loss is observed for the adsorbent. Beyond 450 °C, there is a significant decrease in the mass of Fe_3_O_4_@MOF@PLS, potentially indicating the collapse of the MOF (Figure S3b) and PLS structures. Nevertheless, the adsorbent exhibits robust stability below 450 °C, meeting the requirements for routine detection and analysis.

As exhibited in Figure 3d, the BET surface area and pore volume are 319.7 m^2^/g and 0.47 cm^3^/g, respectively. The pore sizes include 1.5 nm, 2.5 nm, and others. These results suggest that the Fe_3_O_4_@MOF@PLS composite possesses both mesoporous and microporous structures.

**Figure 4:**
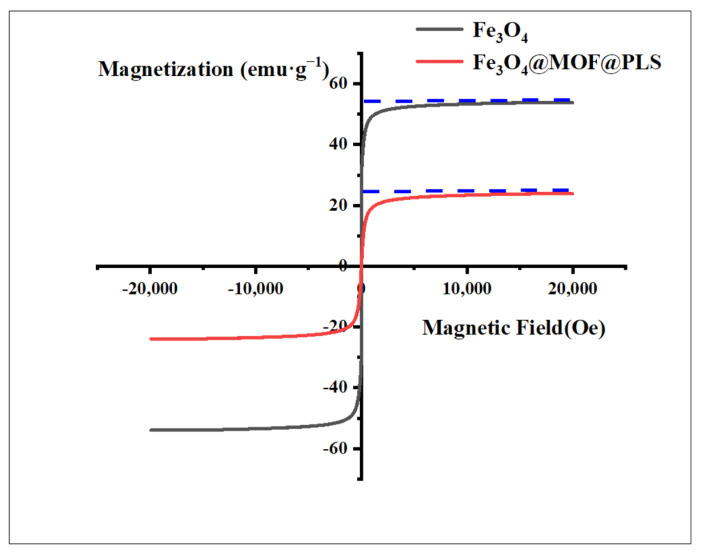
Magnetic strength measurement results of Fe_3_O_4_@MOF@PLS.

VSM was used to measure the magnetic values of Fe_3_O_4_ and Fe_3_O_4_@MOF@PLS, and the results are shown in Figure 4. The measured values of Fe_3_O_4_ and Fe_3_O_4_@MOF@PLS are 53.8 emu/g and 23.2 emu/g, respectively, indicating that the two samples have good magnetic properties. 

### 3.3. Optimization of Chromatographic Conditions

**Figure 5:**
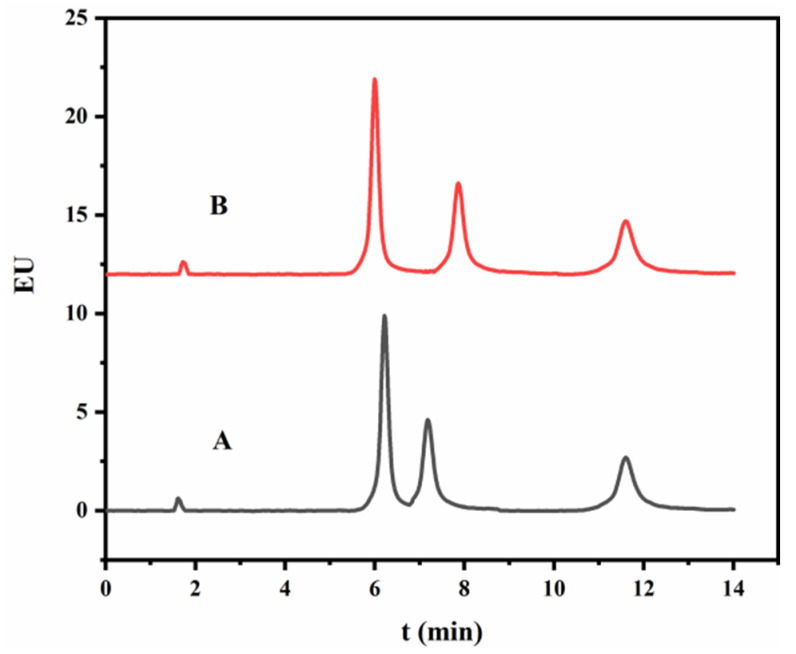
Effects of different flows on the separation degree and signal strength of three antibiotics (A): 0.1% acetic acid water: acetonitrile = 80:20 (*v*:*v*); (B): 0.2% acetic acid water: acetonitrile = 80:20 (*v*:*v*); the three chromatographic peaks from left to right are NFX, CIP, ENR.

The chromatographic conditions were optimized, as shown in Figure 5. The effects of different concentrations of acetic acid on the separation of three FQs under the same volume ratio were investigated. The findings indicate that NFX and CIP do not effectively separate when the mobile phase comprises 0.1% acetic acid water and acetonitrile. However, with a concentration of 0.2% acetic acid, all three FQs successfully separate. As a result, 0.2% acetic acid water: acetonitrile (80:20, *v*:*v*) was chosen as the mobile phase for subsequent experiments.

### 3.4. Optimization of Sample Pretreatment Conditions

In order to obtain the optimal MSPE conditions for NFX, CIP, and ENR, the following factors were optimized: (1) the amount of Fe_3_O_4_@MOF@PLS, (2) sample pH, (3) adsorption time, (4) ionic strength, and (5) elution conditions (including type, volume, and time).

#### 3.4.1. Effect of Fe_3_O_4_@MOF@PLS Dosage

**Figure 6:**
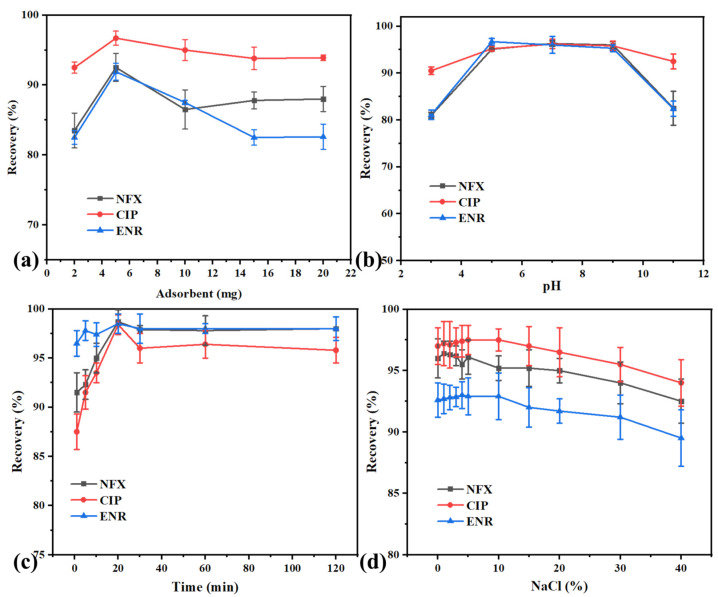
(**a**) Dosage of adsorbent; (**b**) pH; (**c**) adsorption time; (**d**) effect of ionic strength on the recoveries of three antibiotics.

In general, the efficiency of the target analyte during extraction and elution is directly influenced by the amount of adsorbent used. As shown in Figure 6a, when the dosage of adsorbent increases from 2 mg to 5 mg, the extraction efficiency of NFX, CIP, and ENR significantly improve. When the dosage of Fe_3_O_4_@MOF@PLS is 5 mg, the recoveries of the three FQs exceed 90%. However, further increasing the amount of Fe_3_O_4_@MOF@PLS from 5 mg to 20 mg does not significantly enhance recoveries and leads to a slight decrease. This phenomenon may be caused by the saturation state of the adsorbent. Excessive adsorbents may hinder the elution of antibiotics due to their strong adsorption properties, resulting in lower recoveries. Therefore, in order to obtain the highest recovery, a dosage of 5 mg of Fe_3_O_4_@MOF@PLS was determined to be most effective for subsequent optimization processes.

#### 3.4.2. Effect of pH

pH is one of the most important parameters affecting the extraction efficiency of MSPE. It influences both the presence of the analyte and the charge properties of the surface of the MSPE adsorbent. To study the effect of sample pH on the extraction efficiency of NFX, CIP, and ENR, samples were analyzed within a pH range of 3–11. As can be seen from Figure 6b, extraction efficiency varies greatly under different pH conditions. The highest recoveries of NFX, CIP, and ENR are observed when the sample pH values range from 3 to 7, exceeding 95% at pH 7. However, as the pH increases from 7 to 11, the recoveries gradually decline. This trend can be attributed to the varying interactions between adsorbent and analyte. FQs are amphoteric compounds containing both amino and carboxylic groups. The values of pKa_1_ and pKa_2_ are 5.8–6.7 and 7.9–8.9, respectively. In acidic conditions, the amino groups protonate easily, causing NFX, CIP, and ENR to exist as cations, reducing their binding capacity with the adsorbent. Conversely, in alkaline environments, the FQs exist as anions, leading to repulsion between the adsorbent and the analytes, decreasing adsorption capacity. Therefore, in order to obtain maximum recovery, pH of milk samples was adjusted to 7 in subsequent studies.

#### 3.4.3. Effect of Extraction Time

Extraction time is a critical factor in achieving adsorption equilibrium in MSPE. This study examined, the recoveries of three FQs over a range of extraction times from 1 min to 120 min, assessing the impact on the extraction efficiency of FQs. Figure 6c illustrates that the highest recovery is achieved at 20 min, suggesting rapid attainment of extraction equilibrium by the analytes. Consequently, an extraction time of 20 min was chosen for further experiments.

#### 3.4.4. Effect of Ionic Strength

In general, increasing the ionic strength of the sample solution can improve the extraction efficiency through the salting-out effect. However, excessive salt concentrations could reduce extraction efficiency by increasing the viscosity and density of the sample solution. In this study, the effect of ionic strength on extraction efficiency of MSPE was determined by varying the concentration of sodium chloride from 0 to 40%. Figure 6d shows that the recovery of the target analyte remains stable with the concentration of sodium chloride ranging from 0 to 5%, followed by a slight decrease in recovery as the concentration of sodium chloride further increases to 40%. It was observed that higher concentrations of sodium chloride in the sample solution may hinder the adsorption of Fe_3_O_4_@MOF@PLS to three FQs. Given the naturally low ionic strength of the extracted samples, adjustments to the salt concentration of the sample solution during the MSPE process are unnecessary.

#### 3.4.5. Effect of Elution Solvent, Volume, and Time

**Figure 7:**
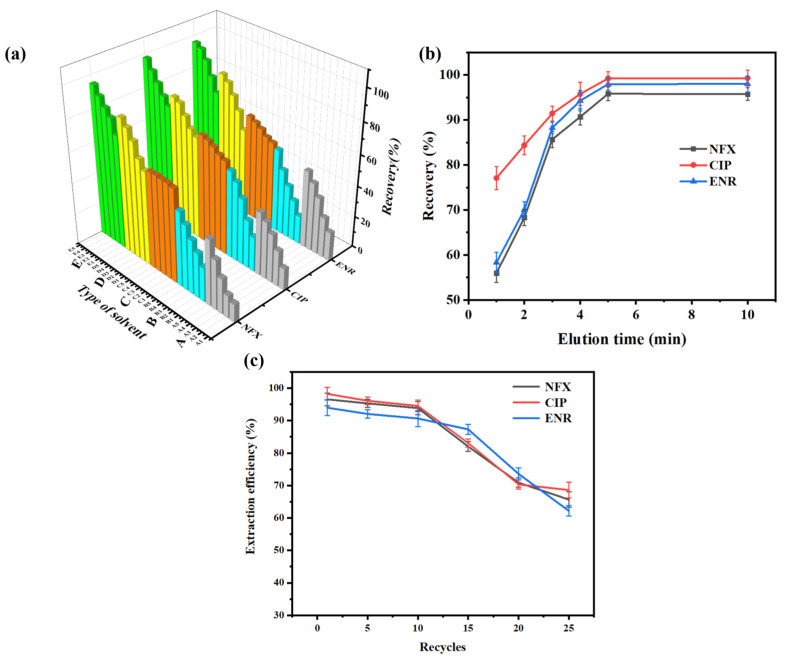
Effects of eluent types (A–E indicate methanol, methanol containing 0.1% acetic acid, acetone, acetonitrile, and acetonitrile containing 0.1% ammonia water, respectively) and eluent volume (the five columns in A–E correspond to 1–5 mL of eluent volume from left to right, For example, A1–A5, respectively) on the recoveries of three FQs (**a**); effect of elution time on the recovery of three FQs (**b**); number of reuses of adsorbents (**c**).

The type and volume of elution solution are the key parameters to ensure accurate quantitative analysis. Firstly, the elution properties of MeOH, 0.1% HAc-MeOH (*v*/*v*), AC, ACN, and 0.1% NH_3_·H_2_O-ACN (*v*/*v*) were evaluated. As shown in Figure 7a, ACN achieves the highest recovery due to its stronger polarity and elution ability compared to MeOH and AC, but it still does not achieve a satisfactory recovery. The most effective recovery can be observed when using 0.1% NH_3_·H_2_O-ACN (*v*/*v*) as the elution solution. The enhanced polarity of the ammonia and acetonitrile mixture likely aids in disrupting the interaction between Fe_3_O_4_@MOF@PLS and the analyte, resulting in recoveries exceeding 90% with a 5 mL eluent volume. Subsequently, the impact of 0.1% NH_3_·H_2_O-ACN (*v*/*v*) on the elution efficiency of the three FQs was evaluated over a period of 1–15 min. As shown in Figure 7b, after 5 min of ultrasonic treatment, the target analytes are almost completely eluted, with recoveries of all three FQs surpassing 95%. Thus, 5 mL of 0.1% NH_3_·H_2_O-ACN (*v*/*v*) ultrasound for 5 min proved to be adequate for eluting the three FQs from Fe_3_O_4_@MOF@PLS.

#### 3.4.6. Reusability

The reusability of Fe_3_O_4_@MOF@PLS was evaluated to assess its regeneration performance. Figure 7c demonstrates that under optimized conditions; the adsorbent could be recycled up to 15 times while maintaining recoveries of over 80% for FQs extracted from milk samples. These results indicate that Fe_3_O_4_@MOF@PLS possesses excellent regeneration capability and can be effectively reused as an MSPE adsorbent. A repeatability experiment comparing different batches of adsorbents was performed. As illustrated in Figure S4, the differences in the adsorption properties of the five batches of Fe_3_O_4_@MOF@PLS, synthesized over five consecutive days for fluoroquinolone antibiotics, are negligible.

### 3.5. Performance of the Proposed Method

#### 3.5.1. Linearity, LOD, and LOQ

To evaluate the efficacy of the established MSPE method for analyzing three FQs in milk samples, various parameters, such as linear range, LOD, LOQ, and precision, were examined under optimized conditions. The results, presented in Table S2, indicate that NFX, CIP, and ENR exhibit a satisfactory linear relationship within the range of 0.5–1000 μg/kg. The linear correlation coefficients (R^2^) for all three FQs surpass 0.9992, indicating good linearity. The LOD and LOQ represent the sensitivity of the instrument, with LOD calculated at three times the signal-to-noise ratio (S/N = 3), and LOQ at ten times the signal-to-noise ratio (S/N = 10). The LOD and LOQ for the three FQs in this study are 0.21–1.33 μg/kg and 0.71–4.42 μg/kg, respectively, which proves that the detection method had high sensitivity.

#### 3.5.2. Recovery and Precision

In this study, the standard concentrations of 5, 25, and 50 μg/kg of three FQs were added into milk samples. The experiments were conducted in quintuplicate to assess the recovery and precision of the established method. The recovery results are shown in Table S3, indicating that the recoveries of the three FQs range from 84.2% to 106.2%. The relative standard deviations (RSDs) are in the range of 3.4–8.8%. These results demonstrate that the method achieves satisfactory recovery, accuracy, and reliability.

#### 3.5.3. Matrix Effect

The matrix effect (ME) plays a crucial role in HPLC analysis, impacting the sensitivity, accuracy, and precision of the method. To evaluate whether the complex matrix in milk samples will affect the determination of FQs, standard solutions (50 μg/L) including three FQs were added to milk and blank solutions for the matrix effect experiment. The experiment was repeated three times. The ME is judged by the percentage of the response value of adding the same amount of analyte to the response value of analyte in pure solvent in the sample matrix according Equation (2). The results, detailed in Table S4 indicate that the matrix effects for NFX, CIP, and ENR are 0.7%, 4.2%, and 2.3%, respectively. It is evident that the matrix effect is negligible, and no matrix correction was deemed necessary.

(2) $$\mathrm{ME}=\frac{B}{A}\;\times \;100\%$$

where A represents the response value of the target in the standard solution and B represents the response value of the target with the same content in the matrix. When −15% ≤ ME ≤ 15%, ME is considered to be insignificant and acceptable. When ME > 15% or ME < −15%, ME is considered to be significant.

### 3.6. Milk Sample Analysis

The practicability of the MSPE-HPLC-FLD method based on Fe_3_O_4_@MOF@PLS was evaluated by detecting the concentrations of three FQs in milk from five local supermarkets, and the detection outcomes are detailed in Table S3. NFX was found in two samples at concentrations of 0.63 μg/kg and 0.49 μg/kg, while ENR was detected in five samples ranging from 1.26 μg/kg to 4.33 μg/kg, CIP was not detected in any of 20 milk samples. 

According to the MRLs of 41 veterinary drugs in Food Safety National Standard Food (GB 31650.1—2022) [43], the MRLs for NFX and ENR in milk are 2 μg/kg and 10 μg/kg, respectively. For adults weighing 60 kg, the daily allowable intake of NFX and ENR should not exceed 120 μg and 600 μg. The highest concentrations of NFX and ENR detected in the samples were 0.63 μg/kg and 4.33 μg/kg, respectively, indicating that the levels of these FQs in the milk were well below the daily allowable intake thresholds, and compliant with the relevant regulations. Nonetheless, there remains a potential risk of dietary exposure, necessitating enhanced management practices and regulatory improvements.

### 3.7. Comparison with Other Methods

In order to evaluate the analytical performance of the established Fe_3_O_4_@MOF@PLS-MSPE-HPLC-FLD method, six indexes, including material dosage, adsorption time, LODs, LOQs, recoveries, and RSDs, were compared to previously reported methods in the literature. The results are summarized in Table 1. Compared to the previously reported analysis method, this approach offers three main advantages. Firstly, it requires a small amount of adsorbent and has a short adsorption time, making it efficient and cost-effective. Secondly, the method demonstrates low LODs and LOQs, indicating high sensitivity. Lastly, it shows improved recoveries and precision, suggesting greater accuracy and reliability. Overall, this method shows promise for the detection and analysis of FQs antibiotics in milk.

## 4. Conclusions

To sum up, a novel magnetic polymer composite Fe_3_O_4_@MOF@PLS was successfully synthesized, displaying stronger adsorption properties compared to PLS and NH_2_-MIL-101 (Fe) in dynamic adsorption experiments. The synthesized Fe_3_O_4_@MOF@PLS composite exhibits excellent magnetic response, good adsorption, and recyclability, making it a promising MSPE adsorbent. Based on the material, a rapid and sensitive MSPE-HPLC-FLD method for the determination of trace FQs residues in milk samples was established by optimizing chromatographic separation conditions and MSPE conditions. Compared with the methods previously reported in the literature, this method offers advantages such as minimal adsorbent usage, low LOD, good linearity, satisfactory recoveries, precision, and so on. The novel method presented here may offer valuable insights and inspiration for detecting trace FQs residues in milk, and further promote the application of magnetic MOF and PLS in complex food matrices for sample pretreatment.

## Figures and Tables

**Table 1:** Comparison of the performance of the proposed method with other reported methods.

Material	Method	Dosage (mg)	Adsorption Time (min)	LODs (μg/kg)	LOQs (μg/kg)	Recoveries (%)	RSDs (%)	Reference
MMMIPs	MSPE-CE -UV	30	20	12.9– 18.8	42.8– 61.9	92.7–97.5	1.8–8.6	[44]
HXLPP- SAX	SPE-HPLC -UV	60	50	2.8–5.1	9.5–17	>85.8	≤9.4	[45]
FPY-COF@ PDA@Fe_3_O_4_	MSPE-HPLC-UV	20	25	2.3–6.8	7.5–22.7	77.8–100.4	<9.7	[24]
M-CCNs @MIP	MSPE-HPLC-DAD	50	63	5.4–12	/	81.2–93.7	2.8–9.8	[46]
MMIP	PMME-HPLC-FLD	/ *	75	0.7	2.3	92.8–95.0	2.4–5.5	[47]
2D MOF	SPE-LC-MS/MS	100	/	0.009–0.016	/	95.0–105.0	<4.21	[48]
Fe_3_O_4_@MOF@PLS	MSPE-HPLC-FLD	5	20	0.21– 1.33	0.71– 4.42	95.4–110.3	1.6–3.6	This work

* “/” represents not mentioned.

## Data Availability

The original contributions presented in the study are included in the article/Supplementary Material, further inquiries can be directed to the corresponding author.

## Supplementary Materials

The following supporting information can be downloaded at: https://www.mdpi.com/article/10.3390/foods13162511/s1, Table S1: Relevant parameters of Langmuir and Freundlich isotherm models for NFX, CIP, and ENR; Table S2: Analytical performance of the proposed method for the determination of FQs; Table S3: Determination of recoveries and concentrations of three antibiotics in milk samples; Table S4: Recovery and matrix effect of the proposed method. Figure S1: Adsorption isotherm fitting of (a) NFX, (b) CIP, and (c) ENR; Figure S2: Adsorption capacities of Fe_3_O_4_@MOF@PLS towards aminoglycosides (gentamicin sulfate and streptomycin sulfate), β-lactams (penicillin sodium and amoxicillin), tetracyclines (terramycin, tetracycline, doxycycline hydrochloride), and FQs (NFX, CIP, ENR, Ofloxacin, and Fleroxacin) and the adsorption capacities of Fe_3_O_4_@PLS towards NFX, CIP, and ENR (dosage of Fe_3_O_4_@MOF@PLS and adsorption capacities of Fe_3_O_4_@PLS: 5 mg; concentration antibiotics solutions: 20 mg/L; volume: 50 mL); Figure S3: (a) FT-IR spectras of Fe_3_O_4_ and PLS; (b) the TGA curves of MOF; Figure S4: Repeatability of different batches of Fe_3_O_4_/MOF/PLS for QN adsorption (dosage of Fe_3_O_4_@MOF@PLS: 5 mg; concentration antibiotics solutions: 20 mg/L; volume: 50 mL).
