# Supplementary material for: Development of Magnetic Porous Polymer Composite for Magnetic Solid Phase Extraction of Three Fluoroquinolones in Milk
Source: Foods. 2024 Aug 12;13(16):2511. doi: 10.3390/foods13162511 (PMC11353990; doi:10.3390/foods13162511)
Supplement: Supplementary file 1 [file foods-13-02511-s001.zip › foods-3121773-supplementary.pdf]

Supplementary material

Development of magnetic porous polymer composite for magnetic solid phase extraction of three fluoroquinolones in milk

Zhendong Yu [1,2,†], Tiantian Xu [1,†], Shumin Lin[1], Shuxuan Liang [1,*]

[1] Key Laboratory of Analytical Science and Technology of Hebei Province, College of Chemistry and Materials Science, Hebei University, Baoding 071002, China
[2] College of Public Health, Hebei University, Baoding 071002, China
# The two authors contributed equally to this work
* Correspondence：liangsx168@126.com

Contents

Table S1…………………………………….S3

Table S2…………………………………….S4

Table S3…………………………………….S5

Table S4…………………………………….S8

Figure S1…………………………………….S9

Figure S2…………………………………….S10

Figure S3…………………………………….S11

Figure S4…………………………………….S12

Table S1. Relevant parameters of Langmuir and Freundlich isotherm models for NFX, CIP, and ENR.

Antibiotic	Temperature (℃)	Langmuir			Freundlich		
		q_m (mg/g)	K_L (L/mg)	R^2	K_F (L/mg)	n	R^2
NFX	30	88.6	9.3×10^{-2}	0.998	21.4	3.1	0.977
	40	98.0	1.1×10^{-1}	0.990	28.3	3.5	0.986
	50	105.0	1.4×10^{-1}	0.992	37.1	4.2	0.984
CIP	30	84.2	8.2×10^{-2}	0.998	19.4	3.1	0.982
	40	86.7	1.2×10^{-1}	0.965	28.2	4.1	0.977
	50	90.7	1.7×10^{-1}	0.975	39.3	5.2	0.973
ENR	30	91.9	1.5×10^{-1}	0.988	36.8	4.8	0.974
	40	96.9	1.9×10^{-1}	0.991	44.0	5.5	0.971
	50	104.0	2.1×10^{-1}	0.976	50.9	6.1	0.971

q_m (mg/g): the adsorption capacity when the adsorbent is fully covered; K_L (L/mg) is the Langmuir adsorption constant; K_F: the Freundlich isotherm constant; n: the heterogeneity factor.

Table S2. Analytical performance of the proposed method for the determination of FQs.

Analyte	Linear range (μg/kg)	Correlation coefficient	LOD (μg/kg)	LOQ (μg/kg)	RSD (%) (n=5)		
					Intra-day	Inter-day	Different batches
NFX	0.5–1000	0.9999	1.33	4.42	3.4	3.7	5.9
CIP	0.5–1000	0.9996	0.40	1.33	1.9	2.6	4.6
ENR	0.5–1000	0.9992	0.21	0.71	2.8	3.7	5.3

Table S3. Determination of recoveries and concentrations of three antibiotics in milk samples.

Milk samples	Added (μg/kg)	NFX		CIP		ENR	
		Found (μg/kg)	Recovery (% ± RSD, n = 3)	Found (μg/kg)	Recovery (% ± RSD, n = 3)	Found (μg/kg)	Recovery (% ± RSD, n = 3)
Milk sample 1	0	ND*		ND		ND	
	5	4.56	91.1 ± 6.7	5.22	104.5 ± 6.1	4.20	84.0 ± 8.7
	25	24.4	97.5 ± 5.4	25.3	101.0 ± 4.4	23.4	93.4 ± 3.7
	50	48.7	91.1 ± 5.8	42.4	84.9 ± 7.2	44.9	89.9 ± 8.9
Milk sample 2	0	ND		ND		ND	
	5	4.42	88.5 ± 4.0	4.40	88.0 ± 8.3	5.20	104.1 ± 6.4
	25	26.2	105.0 ± 5.6	22.1	88.5 ± 6.2	22.6	90.3 ± 4.0
	50	46.1	92.2 ± 4.2	51.8	103.7 ± 7.0	46.9	93.8 ± 5.8
Milk sample 3	0	ND		ND		4.33	
	5	5.20	104.1 ± 6.4	4.90	97.9 ± 6.9	5.10	101.9 ± 8..8
	25	22.6	90.3 ± 4.0	25.1	100.2 ± 3.4	24.6	98.2 ± 7.5
	50	46.9	93.8 ± 5.8	52.5	105.0 ± 4.1	43.4	86.9 ± 3.4
Milk sample 4	0	0.63		ND		ND	
	5	4.91	98.3 ± 7.2	4.50	90.0 ± 4.3	4.86	97.2 ± 4.7
	25	24.4	97.7 ± 4.6	20.2	85.5 ± 4.4	23.3	93.2 ± 5.5
	50	48.6	97.3 ± 7.1	47.5	95.8 ± 4.9	43.6	87.3 ± 5.3
Milk sample 5	0	ND		ND		ND	
	5	4.68	93.6 ± 5.2	4.31	86.3 ± 4.3	4.60	91.9 ± 5.5
	25	24.1	96.2 ± 8.8	24.0	96.0 ± 4.1	23.7	95.1 ± 8.1
	50	47.1	94.1 ± 6.9	50.5	101.1 ± 4.1	44.8	89.6 ± 4.7
Milk sample 6	0	ND		ND		2.78	
	5	4.42	88.3 ± 8.7	4.30	85.9 ± 5.8	5.03	100.7 ± 6.2
	25	22.0	88.1 ± 5.0	24.3	97.3 ± 6.1	23.9	95.4 ± 7.2
	50	44.4	88.9 ± 5.7	49.0	98.0 ± 6.9	42.2	84.3 ± 8.6
Milk sample 7	0	0.49		ND		ND	
	5	4.67	93.4 ± 6.9	5.16	103.2 ± 4.0	4.44	88.9 ± 3.8
	25	23.6	94.5 ± 8.3	22.3	89.1 ± 4.5	23.5	94.0 ± 5.2
	50	44.8	89.6 ± 5.6	47.2	94.4 ± 6.3	50.1	100.1 ± 7.1
Milk sample 8	0	ND		ND		4.13	
	5	4.43	88.5 ± 5.8	4.43	88.6 ± 7.5	4.70	94.0 ± 3.6
	25	21.8	87.2 ± 5.4	22.9	91.5 ± 3.4	26.1	104.2 ± 6.8
	50	47.2	94.5 ± 8.5	44.6	89.3 ± 4.5	46.4	92.9 ± 6.1
Milk sample 9	0	ND		ND		ND	
	5	4.21	84.2 ± 8.7	4.56	91.2 ± 6.9	4.70	94.0 ± 4.6
	25	21.8	87.4 ± 3.9	23.6	94.3 ± 4.8	24.7	98.8 ± 3.4
	50	44.7	89.5 ± 7.9	42.2	84.4 ± 5.0	49.8	99.7 ± 7.2
	0	ND		ND		ND	
	5	4.77	95.4± 7.9	4.96	99.1 ± 4.3	4.29	85.7 ± 7.3

Milk samples	Added (μg/kg)	NFX		CIP		ENR	
		Found (μg/kg)	Recovery (% ± RSD, n = 3)	Found (μg/kg)	Recovery (% ± RSD, n = 3)	Found (μg/kg)	Recovery (% ± RSD, n = 3)
Milk sample 9	25	22.9	91.6± 5.9	21.7	87.0 ± 8.1	23.4	97.5 ± 5.4
	50	50	51.1	47.3	94.6 ± 6.4	48.0	96.0 ± 7.0
	0	ND		ND		2.72	
Milk sample 11	5	4.70	93.6 ± 3.9	5.01	100.3 ± 6.6	4.65	92.9 ± 4.0
	25	23.6	94.3 ± 6.8	22.4	89.6 ± 6.4	25.9	103.5 ± 3.8
	50	43.0	86.0 ± 7.3	48.4	96.9 ± 7.0	42.7	85.4 ± 3.3
	0	ND		ND		ND	
Milk sample 12	5	4.58	91.6 ± 4.2	4.52	90.5 ± 4.1	4.90	98.0 ± 5.9
	25	24.4	97.8 ± 4.6	23.1	92.5 ± 7.2	21.1	84.5 ± 4.3
	50	49.5	99.1 ± 6.6	45.5	91.1 ± 7.9	51.4	102.9 ± 7.4
	0	ND		ND		1.26	
Milk sample 13	5	4.55	91.0 ± 7.1	5.05	101.1 ± 8.1	4.85	96.8 ± 8.5
	25	24.4	97.8 ± 8.5	23.7	94.8 ± 8.9	23.3	93.4 ± 8.2
	50	53.1	106.2 ± 4.8	49.8	99.6 ± 4.2	51.9	103.8 ± 4.3
	0	ND		ND		ND	
Milk sample 14	5	4.35	86.8 ± 7.6	4.46	89.2 ± 7.4	5.10	102.2 ± 7.2
	25	21.7	86.9 ± 7.8	24.2	96.9 ± 3.8	24.4	97.5 ± 7.6
	50	49.7	99.4 ± 8.7	45.4	90.9 ± 4.5	44.2	88.3 ± 5.0
	0	ND		ND		ND	
Milk sample 15	5	4.58	91.6 ± 5.6	5.28	105.6 ± 7.9	0.88	88.1 ± 3.7
	25	23.1	92.2 ± 8.9	21.2	84.9 ± 3.9	24.6	98.3 ± 4.3
	50	44.8	89.6 ± 6.1	43.6	87.3 ± 6.5	43.6	87.2 ± 4.8
	0	ND		ND		ND	
Milk sample 16	5	4.55	91.0 ± 7.1	5.05	101.1 ± 8.1	4.85	96.8 ± 8.5
	25	24.4	97.8 ± 8.5	23.7	94.8 ± 8.9	23.3	93.4 ± 8.2
	50	53.1	106.2 ± 4.8	49.8	99.6 ± 4.2	51.9	103.8 ± 4.3
	0	ND		ND		ND	
Milk sample 17	5	4.35	86.8 ± 7.6	4.46	89.2 ± 7.4	5.10	102.2 ± 7.2
	25	21.7	86.9 ± 7.8	24.2	96.9 ± 3.8	24.4	97.5 ± 7.6
	50	49.7	99.4 ± 8.7	45.4	90.9 ± 4.5	44.2	88.3 ± 5.0
	0	ND		ND		ND	
Milk sample 18	5	4.58	91.6 ± 5.6	5.28	105.6 ± 7.9	4.41	88.1 ± 3.7
	25	23.1	92.2 ± 8.9	21.2	84.9 ± 3.9	24.6	98.3 ± 4.3
	50	44.8	89.6 ± 6.1	43.6	87.3 ± 6.5	43.6	87.2 ± 4.8
	0	ND		ND		ND	
Milk sample 19	5	4.75	95.4 ± 9.0	4.71	93.6 ± 3.9	4.57	91.6 ± 4.2
	25	22.9	91.4 ± 5.7	23.6	94.3 ± 6.8	24.4	97.8 ± 4.6
	50	51.1	102.2 ± 3.6	43.0	86.0 ± 7.3	49.6	99.1 ± 6.6
	0	ND		ND		ND	

Milk samples	Added (μg/kg)	NFX		CIP		ENR	
		Found (μg/kg)	Recovery (% ± RSD, n = 3)	Found (μg/kg)	Recovery (% ± RSD, n = 3)	Found (μg/kg)	Recovery (% ± RSD, n = 3)
Milk sample 20	5	4.29	85.7 ± 7.3	4.65	92.9 ± 4.0	4.90	98.0 ± 5.9
	25	23.4	97.5 ± 5.4	25.9	103.5 ± 3.8	21.3	84.5 ± 4.3
	50	48.0	96.0 ± 7.0	42.7	85.4 ± 3.3	51.3	102.9 ± 7.4

* Not detected

Table S4. Recovery and matrix effect of the proposed method.

Addition concentration (μg/kg)	Analyte	Recovery (%)		ME [a]±RSD (%)
		Standard solution	Milk sample	
50	NFX	98.6	97.9	0.7±2.4
	CIP	109.7	105.5	4.2±1.6
	ENR	102.6	100.3	2.3±1.8

[a] Matrix effect

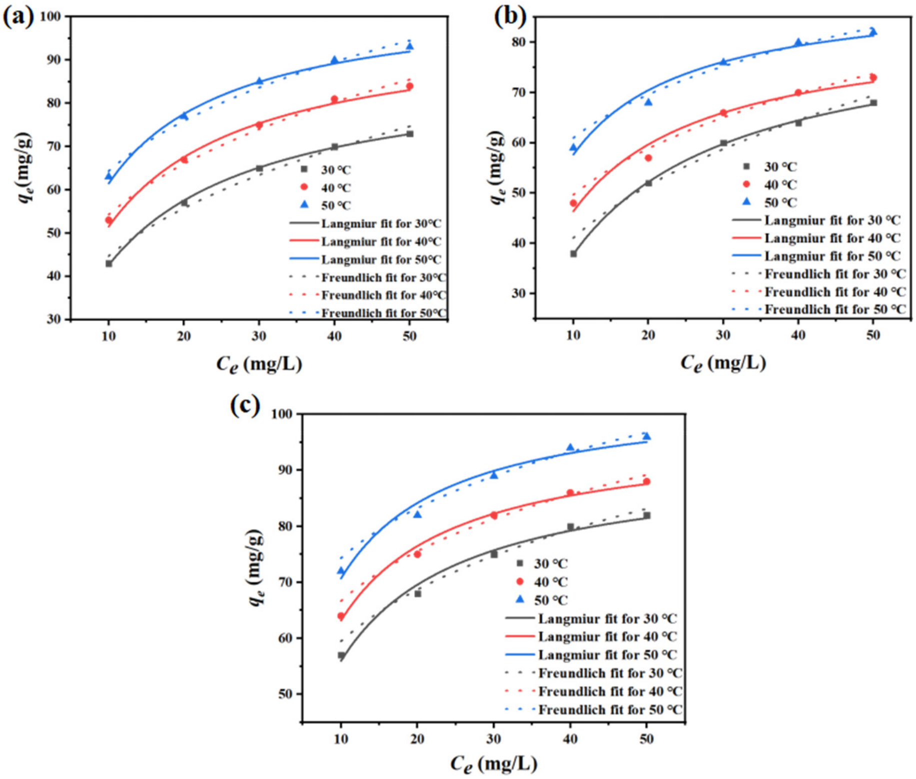

Figure S1. Adsorption isotherms fitting of (a) NFX, (b) CIP, and (c) ENR.

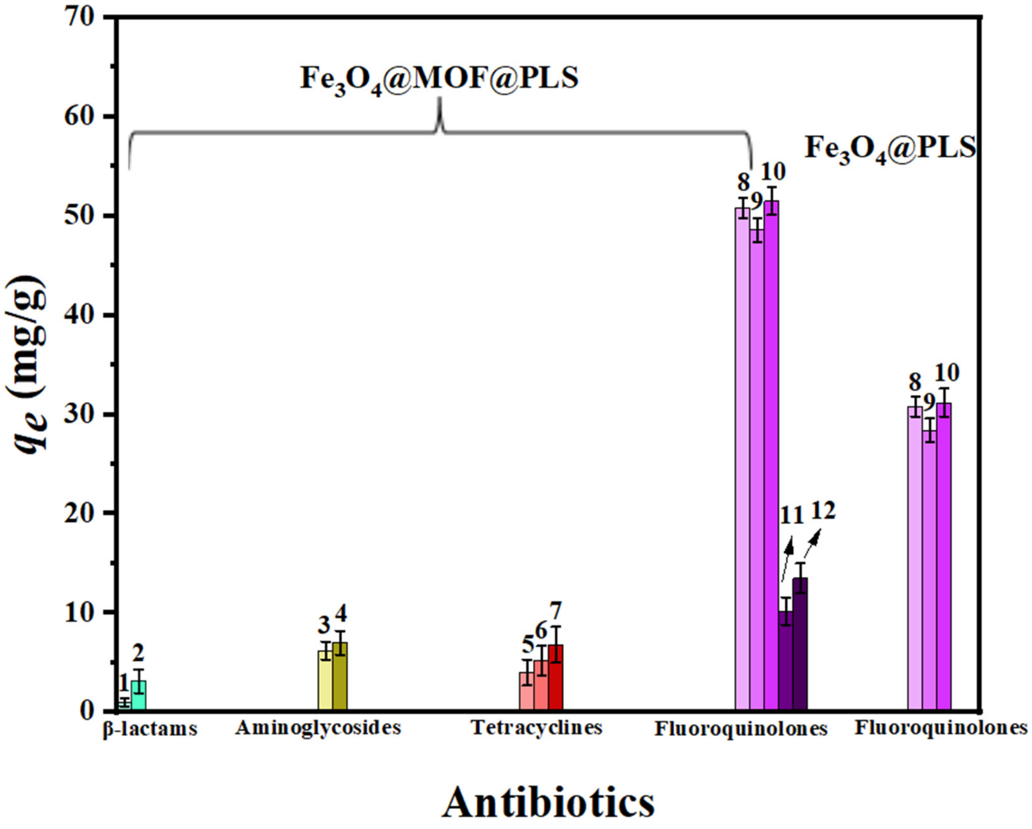

Figure S2. Comparison of the adsorption capacities of four types of antibiotics including aminoglycosides (1-gentamicin sulfate, 2-streptomycin sulfate), β-lactams (3-penicillin sodium, 4-amoxicillin), tetracyclines (5-terramycin, 6-tetracycline, 7-doxycycline hydrochloride) and FQs (8-NFX, 9-CIP, 10-ENR, 11-ofloxacin and 12-fleroxacin) on Fe_3O_4@MOF@PLS and adsorption capacities of Fe_3O_4@PLS (dosage of Fe_3O_4@MOF@PLS: 5 mg; concentration antibiotic solutions: 20 mg/L; volume: 50 mL).

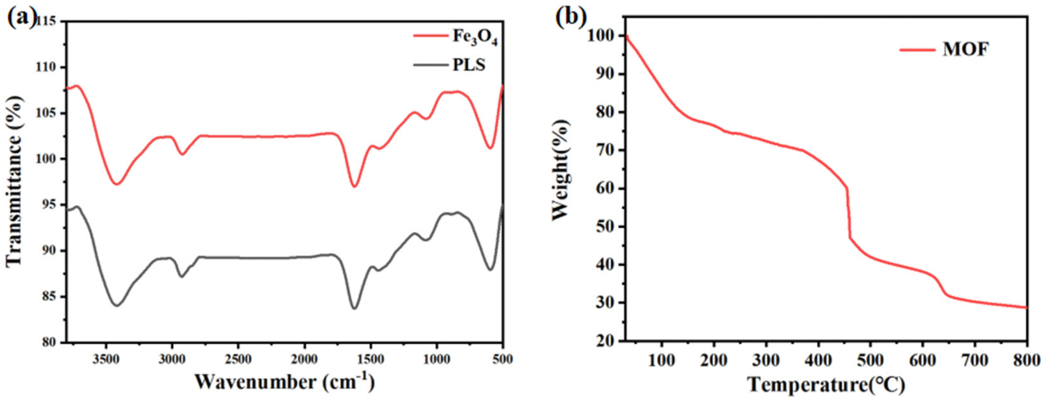

Figure S3. (a) FT-IR spectrograms of Fe_3O_4 and PLS; (b) TGA curves of MOF.

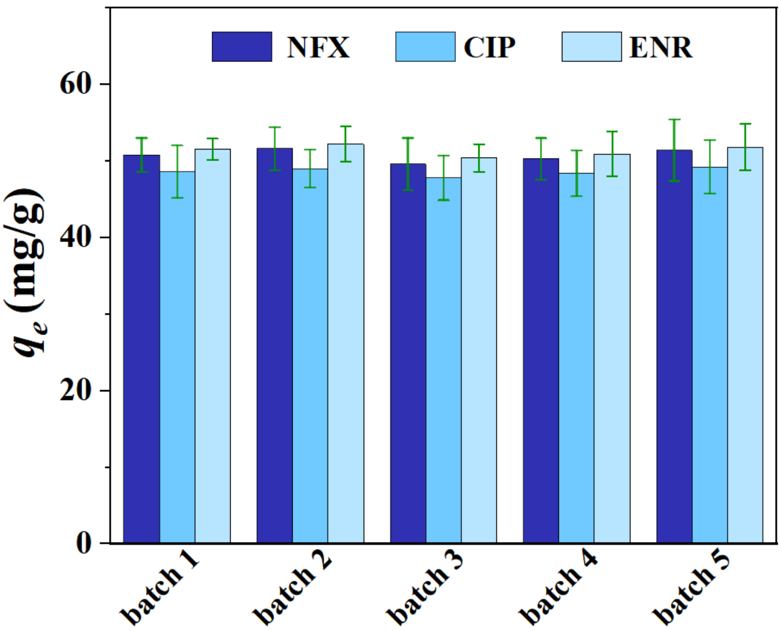

Figure S4. Repeatability of different batches of Fe_3O_4/MOF/PLS for QNs adsorption (dosage of Fe_3O_4@MOF@PLS: 5 mg; concentration antibiotics solutions: 20 mg/L; volume: 50 mL).
